# A novel methodology for personalized simulations of ventricular hemodynamics from noninvasive imaging data

**DOI:** 10.1016/j.compmedimag.2016.03.004

**Published:** 2016-07

**Authors:** A. de Vecchi, A. Gomez, K. Pushparajah, T. Schaeffter, J.M. Simpson, R. Razavi, G.P. Penney, N.P. Smith, D.A. Nordsletten

**Affiliations:** a Division of Imaging Sciences and Biomedical Engineering, King’s College London, St. Thomas’ Hospital, London SE1 7EH, UK; b Evelina London Children’s Hospital, London SE1 7EH, UK

**Keywords:** Cardiac hemodynamics, Personalized computational modeling, 3D blood flow reconstruction, B-Mode and Color Doppler echocardiography, PC-MRI

## Abstract

Current state-of-the-art imaging techniques can provide quantitative information to characterize ventricular function within the limits of the spatiotemporal resolution achievable in a realistic acquisition time. These imaging data can be used to personalize computer models, which in turn can help treatment planning by quantifying biomarkers that cannot be directly imaged, such as flow energy, shear stress and pressure gradients. To date, computer models have typically relied on invasive pressure measurements to be made patient-specific. When these data are not available, the scope and validity of the models are limited. To address this problem, we propose a new methodology for modeling patient-specific hemodynamics based exclusively on noninvasive velocity and anatomical data from 3D+t echocardiography or Magnetic Resonance Imaging (MRI). Numerical simulations of the cardiac cycle are driven by the image-derived velocities prescribed at the model boundaries using a penalty method that recovers a physical solution by minimizing the energy imparted to the system. This numerical approach circumvents the mathematical challenges due to the poor conditioning that arises from the imposition of boundary conditions on velocity only. We demonstrate that through this technique we are able to reconstruct given flow fields using Dirichlet only conditions. We also perform a sensitivity analysis to investigate the accuracy of this approach for different images with varying spatiotemporal resolution. Finally, we examine the influence of noise on the computed result, showing robustness to unbiased noise with an average error in the simulated velocity approximately 7% for a typical voxel size of 2 mm^3^ and temporal resolution of 30 ms. The methodology is eventually applied to a patient case to highlight the potential for a direct clinical translation.

## Highlights

• A new imaging-modeling methodology to simulate ventricular hemodynamics is proposed.
• The models are solely based on noninvasive data from MRI or echocardiography.
• The workflow is patient-specific and time-efficient for direct clinical application.
• The models can quantify hemodynamic metrics and relative pressure with high resolution.
• The methodology is robust to noise and low resolution in the imaging data.

## Graphical abstract

**Figure:**
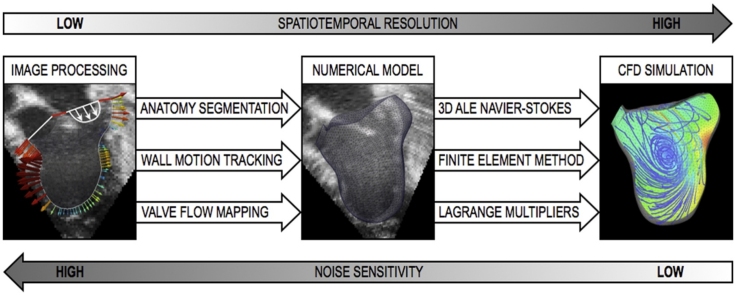

## 1 Introduction

Cardiac pathologies often show a high interindividual variability in both the anatomy and the response to treatment, making population-based metrics less effective in defining therapy. A patient-specific approach is therefore crucial for successfully evaluating the pump function in the diseased heart and customizing treatment to the patient’s pathophysiology. Recent developments in clinical imaging have underpinned the value of personalized medicine as a powerful alternative to traditional healthcare. For example, blood flow velocity and direction can now be quantified noninvasively from both 3D+t echocardiography and Phase-Contrast MRI (PC-MRI) (Gatehouse et al., 2005, Gomez et al., 2015, Gomez et al., 2013b). Metrics derived from these hemodynamics parameters, such as the diastolic vortex formation, have been recognized as indicators of cardiac performance, placing emphasis on the importance of the intraventricular blood flow patterns (Pedrizzetti et al., 2014, Sengupta et al., 2012). Intraventricular flow propagation speed can also be used to estimate left ventricular filling pressures and to evaluate diastolic function (Bruch et al., 2005, Firstenberg et al., 2000, Greenberg et al., 2001, Nagueh et al., 2009). Similarly, ejection parameters and blood kinetic energy obtained from both PC-MRI and 3D+t echocardiography by flow mapping can yield information on the cardiac energetic efficiency (Markl et al., 2011, Yang et al., 2007), and techniques of wall motion tracking are routinely used to infer the distribution of strains and their rate. These are generally based on tagged MRI data (Axel et al., 2005, Chandrashekara et al., 2004, Pan et al., 2005); however, algorithms have also been developed for 3D+t echocardiography and standard MRI Cine sequences (Papademetris et al., 2001, Shi et al., 2013). Despite this progress, however, the accuracy of measurement depends on the temporal and spatial resolution of the images, which is dictated by technical limitations and by the necessity to keep the acquisition times at a minimum. The errors associated with low spatiotemporal resolution are particularly significant when derivatives of velocity are used to quantify metrics such as the power loss in the blood flow, or in the derivation of relative pressures from the reconstructed velocity field (Lamata et al., 2013, Yotti et al., 2004). Metrics derived from echocardiographic data have also been proved unable to track reliably ventricular pressure variations as a result of treatment within individual subjects (Bhella et al., 2011).

In this context, a synergy between clinical imaging and computer modeling has the potential to provide accurate patient-specific information to assist the clinical decision-making process. Recently, computational modeling has reached a stage of development with capability to simulate cardiac function realistically and to augment the traditional clinical approaches (McCormick et al., 2013, Tang et al., 2010). However, this potential is currently not fully exploited, as model personalization often relies on invasive pressure data acquired through catheterization, which in complex pathologies is limited by technical difficulties and risks for the patient, and on detailed information on the orientation of the myocardial fiber architecture. These factors currently limit the extent to which this technique can be effectively used for individual treatment planning. The main challenge lies therefore in the capability to personalize the models accurately with the least number of assumptions, given the available clinical data. Thanks to their very high spatial and temporal resolution, patient-specific models can provide an accurate quantification of velocity-based metrics and pressure gradients, and thus complement the information from imaging data. Specifically, the value of the approach resides in the ability to make use of the most reliable image-derived information available to build patient-specific models that, in turn, can quantify metrics that cannot be directly derived from the imaging data, such as pressure gradients and flow energy. Where a full description of myocardial properties and pressures is not available, computational fluid dynamics (CFD) simulations of ventricular hemodynamics can be driven by velocities extracted from time-resolved image sequences. In this context, the ability to personalize the models solely based on velocity data would result in a more robust and time-efficient modeling strategy, with the additional advantage of not relying on invasive or non-standard measurements.

This modeling strategy is promising but has a number of mathematical and numerical challenges. Even where low-noise imaging data are available, the exclusive imposition of velocity values (via Dirichlet boundary conditions) leads to a non-unique pressure solution and to a potential violation of mass conservation in the Navier–Stokes problem. For these reasons, commonly used models for ventricular flow tend to prescribe Dirichlet conditions on only a portion of the domain boundary. Traction or pressure conditions are then imposed on the valve planes in the attempt to recover an inlet or outlet velocity profile similar to that reconstructed from the images (Saber et al., 2003, Schenkel et al., 2009 Schenkel et al., 2009). In a different approach, the problem of mass conservation is tackled by imposing a hybrid boundary condition, consisting of a Dirichlet condition on velocity and a Neumann condition on pressure, where the derivative of the variable is constrained instead to its direct value. This hybrid condition is then prescribed on separate patches nested in the valve plane (Long et al., 2008, Long et al., 2003). However, the definition of a pressure boundary of arbitrary size, value and position within the inlet plane is still not ideal, as it can lead to alterations in the valve velocity profile since the velocity inside the pressure patch cannot be controlled directly. Other studies propose a Lagrange multiplier technique to impose the actual flux at the inflow and outflow of vessels, where the boundary conditions are applied in a weak sense (Veneziani and Vergara, 2007, Veneziani and Vergara, 2005). This method has also been extended to solve the Navier–Stokes equations with an additional constraint on the minimization of the difference between the computed and the given flow rate at a boundary (Formaggia et al., 2010, Formaggia et al., 2008). More recently, a different numerical approach to image-based CFD simulations has been presented (Chnafa et al., 2014, Chnafa et al., 2012). Here, an ALE formulation of the incompressible Navier–Stokes equations is solved using a fourth order finite-volume technique coupled with a Large Eddies Simulation (LES) turbulence model. In this method the valves are modeled using an immersed boundary method.

The present numerical implementation proposes an alternative methodology for solving the problem of driving flow within ALE Navier–Stokes simulations with finite elements. The novelty of this technique resides in the application of penalty methods to image-based modeling. Specifically, the penalty term is introduced in the weak form of the Dirichlet boundary conditions on the velocity derived from imaging data so that global mass conservation is achieved. This is the first time that this technique is applied to achieve realistic ventricular simulations. To accelerate the model generation, the numerical solver is also fully integrated with the image processing workflow. Further, to broaden the applicability range of the method, this toolbox has been designed to be compatible with different imaging modalities, including 3D echocardiography and MRI. The methodology is first implemented and verified in a 2D idealized ventricle model with different levels of noise. The workflow is then applied to model the full cardiac cycle in the left ventricle of a pediatric patient. To test the robustness of the approach to variations in the image quality, synthetic imaging data with different combination of temporal and spatial resolution are generated from the same patient dataset. The workflow to extract the velocity from the image sequences is then performed for each combination of spatial and temporal resolution and the corresponding boundary conditions are used to perform the simulations in each case. The *L*^2^ error between the original and the down-sampled results is presented along with clinical metrics such as the blood kinetic energy and the intraventricular pressure gradients to demonstrate the potential for clinical applicability.

## 2 Methods

The integrated imaging-modeling workflow for the numerical simulations consists of four steps: (i) image acquisition (3D+t echocardiography, Cine MR, PC-MRI sequences); (ii) generation of a patient-specific volume mesh of the ventricular cavity at end-systole; (iii) derivation of velocity boundary conditions from imaging data (wall motion tracking and valvular flow mapping); (iv) numerical simulations of ventricular hemodynamics during the cardiac cycle. This is achieved by solving an augmented formulation of the full 3D ALE Navier–Stokes problem for incompressible flows by means of the Galerkin finite element method.

The proposed methodology can be applied to both MRI and echocardiography data, as the wall motion tracking and the 3D flow mapping algorithms are not restricted to a specific imaging modality. The following sections thus describe the general modeling workflow regardless to the type of the imaging datasets used. The patient model presented in this study, however, is based on 3D+t echocardiography data, as described in more detail in Section 3.2.1.

### 2.1 Image acquisition and processing

**Fig. 1:**
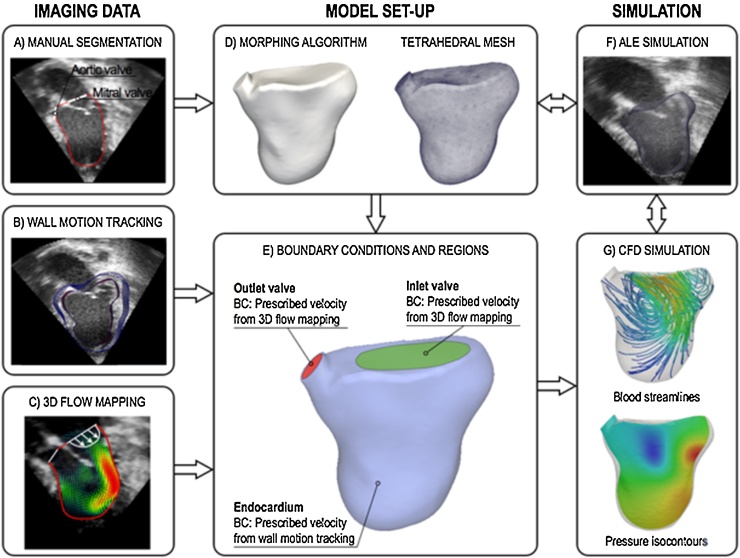
Diagram of the methodological framework, consisting of image processing (A–C), model generation (D–E) and numerical simulations (F–G).

The proposed methodology uses a wall motion tracking algorithm based on Temporal-Sparse Free Form Deformations (TSFFD), which has been validated for both MR and echocardiography data and is able to capture the full 3D motion field (Shi et al., 2013). A technique developed by our group is used to reconstruct the 3D flow field from spatiotemporal imaging sequences such as 3D+t echocardiography or PC-MRI (Gomez et al., 2013a, Gomez et al., 2013b). This method estimates a continuous, derivable, diffeomorphic and periodic motion field using a multi-scale sparse B-spline registration. The resulting motion field is then applied to propagate a pre-segmented surface mesh of the left ventricle in end systole throughout the cardiac cycle. Cubic interpolation is used to create a propagated mesh every millisecond. To enhance the scope and flexibility of the workflow, the algorithm for wall motion tracking can be directly applied to standard imaging data such as stack Cine MRI series or 3D BMode echocardiography, as opposed to wall motion tracking from tagged MRI, which requires dedicated acquisition protocols. Similarly, the 3D flow mapping can be reconstructed from Color Doppler echocardiography data where more sophisticated imaging techniques like PC-MRI are not available due to technical difficulties (e.g., presence of non MR-compatible implants, long acquisition times necessary to achieve a sufficient spatiotemporal resolution in small hearts etc.,). Anatomical segmentation, wall motion and valvular flow mapping provide the necessary information for the model set-up and validation, as shown in Fig. 1A–C. In the patient case, the 3D+t echocardiography sequence was acquired in the left ventricle using a Philips X3-1 matrix array cardiac probe. A 4-beat acquisition with temporal resolution of 16 time frames per cardiac cycle was used with a spatial resolution of 0.5 × 0.5 × 0.5 mm. Full spatial coverage of the cavity was achieved.

### 2.2 Mesh generation

The patient’s anatomy is obtained by manually segmenting the ventricular cavity and the position of the valve planes at end-systole from the 3D image data. A triangular surface mesh is morphed to the segmented anatomy (Fig. 1D) using a fitting algorithm that iteratively deforms a template half-ellipsoidal mesh until the desired contours are achieved (Lamata et al., 2011). In the final step, a volume mesh with unstructured tetrahedral elements is generated from the deformed surface mesh using the software package *Cubit* (Sandia National Laboratories, New Mexico, USA). Three boundary patches, i.e. endocardium, inlet and outlet valve, are subsequently identified based on manual segmentation landmarks (Fig. 1E). A simple ALE simulation driven by the prescribed wall motion is performed to test the robustness of the mesh to localised deformations (Fig. 1F) (Nordsletten et al., 2010b). This ensures that no significant deterioration of the elements quality occurs during the complete hemodynamic simulation of the cardiac cycle. The mesh quality is improved until the aspect ratio of each tetrahedron is above a value of 0.35, where quality is measured using the radius ratio (Liu and Joe, 1994).

### 2.3 Imposition of boundary conditions

The correct derivation of the boundary conditions from the imaging data is a crucial step for the modeling accuracy. In the patient case, the motion of the endocardium and valve planes was extracted from the 3D+t BMode sequences. These images have a speckle texture that moves with anatomy, which is non-arbitrary and therefore allows us to capture all components of the velocity, without neglecting the tangential contribution. This velocity derived from the imaging data, ***v_d_***, is then prescribed as a Dirichlet boundary condition on the flow domain Ω in the models. During inflation (diastole), the velocity in the outflow tract is set to be that of the valve plane itself and an inflow velocity profile is prescribed at the inlet plane consistently with the volume change, calculated from the previously computed motion field. Similarly, in contraction (systole) the inlet valve remains closed and moves according to the valve plane motion detected from the imaging data. This technique takes therefore into account the valve plane motion, and associated velocity, during the cardiac cycle, which is a biomarker of clinical importance (Ommen and Nishimura, 2003). The ventricular wall motion and the inlet/outlet velocity vectors reconstructed from the image sequences are first interpolated to a time resolution of 1 ms, which is required for the numerical stability of the Navier–Stokes solver. This process is achieved by cubic interpolation and its accuracy depends on the initial temporal resolution of the data. Spatial interpolation of the wall motion vectors is directly provided by the TSFFD algorithm using B-spline interpolation. These Dirichlet boundary conditions are then used to drive patient-specific simulations of ventricular hemodynamics by solving the incompressible 3D ALE Navier–Stokes system (Fig. 1G).

### 2.4 Numerical solution of the Navier–Stokes boundary value problem

#### 2.4.1 Weakly imposed Dirichlet problem using Lagrange multipliers

The proposed workflow is based exclusively on noninvasive velocity data and does not rely on information on the absolute pressure in the ventricle. When all boundary conditions are specified on the velocity, the pressure solution becomes unique only up to a constant, which may be arbitrary selected by prescribing pressure at a node. This Dirichlet condition on the pressure removes arbitrary constants, ensuring unique pressure by removing constants from the test space. In theory, this means the weak form of mass conservation no longer ensures global mass conservation; however, for Navier–Stokes, this is usually guaranteed by the compatibility condition, which must hold on the boundary data (Quarteroni and Valli, 2008). While this process is straightforward in the solution of Navier–Stokes, it is more cumbersome in ALE Navier–Stokes, as the ALE equivalent of the compatibility conditions requires consistency in the boundary data being divergence free over the moving domain in a manner that is equivalent to the ALE discretization scheme used. This is challenging to compensate for, given the inevitable inconsistencies in the image-derived domain motion coming from noise or processing. As a result, any error in the ALE variant of the compatibility condition when driving the flow via Dirichlet boundary conditions results in a violation of global mass conservation that is ‘lost’ or ‘gained’ at the selected pressure node.

To recover a physical solution with a set of Dirichlet velocity boundary conditions, we propose a technique that maintains robustness along with well-posedness of the boundary value problem. The stabilization is achieved by adding an extra term to the conservation of momentum in the Navier–Stokes system to minimize the energy of the fluid at the boundary where the constraint on the flow velocity is imposed. To illustrate the basic mathematical principles behind the full Navier–Stokes formulation solved in our approach, see Eqs. (5)–(7), we first consider the Dirichlet problem of Stokes flow. The velocity extracted from the flow reconstruction at the ventricular inlet and outlet, and the wall motion tracking in the 3D+t imaging data is the known variable $v_{d}$ and is imposed on the corresponding boundaries Γ of the flow domain Ω (i.e. the ventricular blood pool) via the condition $v=v_{d}$ on $\Gamma_{\Omega }$. The velocity and pressure (v,p) of the blood inside the ventricle, which describe the blood pool dynamics in space and time, can be written as the critical point of an energy functional Π (Brooks and Hughes, 1982), i.e.,

(1) $$\begin{array}{cc} \Pi \left(v,\;p\right)=\underset{u\in H_{D}^{1}\left(\Omega \right)}{\inf}\underset{q\in L^{2}(\Omega )\backslash \mathbb{R}}{\sup}\Pi \left(u\;,q\right), & H_{D}^{1}\left(\Omega \right)=\left\{u\in H^{1}\left(\Omega \right)|u=v_{d}\;\;\;\;\;on\;\;\;\;\;\Gamma_{\Omega }\right\} \end{array}$$

where $\Pi (u,q)=\int_{\Omega }\mu |\nabla_{x}u{|}^{2}+q\nabla_{x}\cdot u\text{d}\Omega$, μ is the flow viscosity, and $H^{1}\left(\Omega \right)$ and $L^{2}\left(\Omega \right)$ are Hilbert spaces (Quarteroni and Valli, 2008). An alternative form is to augment the energy functional in Eq. (1), with a boundary term (Bercovier, 1978), i.e.,

(2) $$\Pi_{\lambda }(u,q,l)=\Pi (u,q)+\int_{\Gamma_{v}}l\cdot \left(u-v_{d}-\frac{1}{2k}l\right)\text{d}\Gamma$$

where an additional variable ***l*** is added along a portion of the Dirichlet boundary Γ_*v*_ (e.g. the valve inlet or outlet) to make the solution satisfy the velocity data ***v_d_*** derived from the images at that specific boundary in a relaxed sense. The accuracy with which this data is imposed is governed by the parameter *k* *>* *0*. In this case, assuming that 1/*k* > 0 on at least a portion of Γ_*v*_, our Stokes solution (***v***, *p*, ***λ***) is that which minimises the viscous energy, maximises the hydraulic energy and maximises the boundary term, i.e.,

(3) $$\Pi_{\lambda }\left(v,p,\lambda \right)=\underset{u\in H_{D\backslash \Gamma_{v}}^{1}\left(\Omega \right)}{\inf}\underset{\left(q,l\right)\in L^{2}\left(\Omega \right)\times L^{2}\left(\Gamma \right)}{\sup}\Pi_{\lambda }\left(u,q,l\right),$$

with $H_{D\backslash \Gamma_{v}}^{1}\left(\Omega \right)=\left\{u\in H^{1}\left(\Omega \right)|u=v_{d}\;\;\;\;\;on\;\;\;\;\;\Gamma \backslash \Gamma_{v}\right\}$. Conceptually, ***λ*** represents the boundary traction on $\Gamma_{v}$ and the additional term represents a balance on the power imparted to the system modulated by the parameter k (with units Ns/m). As k→∞, the solution approaches the full Dirichlet problem, whereby an infinite amount of power will be generated, if necessary, to allow the Stokes solution to match the velocity from the data, $v_{d}$. However, in this case the energy functional goes unbounded if $v_{d}$ violates the compatibility condition, as we then have competing maximisation principles. In contrast, as k→0, the solution effectively ignores the boundary data and sends λ→0. In the case where, 1/k=0 over some (but not all) of the boundary $\Gamma_{v}$, it is noted that λ is a Lagrange multiplier and relies on the inf-sup condition,

(4) $$\begin{array}{cc} \underset{u\in H_{0\backslash \Gamma_{v}}^{1}(\Omega )}{\sup}\frac{\int_{\Omega }q\nabla_{x}\cdot ud\Omega +\int_{\Gamma_{v}}l\cdot ud\Gamma }{{\left\|u\right\|}_{1}}\geq \beta {\left({\left\|q\right\|}_{0}^{2}+{\left\|l\right\|}_{0,\Gamma_{v}}^{2}\right)}^{\frac{1}{2}} & \forall (q,l)\in L^{2}(\Omega )\times L^{2}(\Gamma ). \end{array}$$

with $H_{0\backslash \Gamma_{v}}^{1}\left(\Omega \right)$ denoting the subspace of $H^{1}\left(\Omega \right)$ with functions set to zero on $\Gamma \backslash \Gamma_{v}$ and ${∥\cdot ∥}_{1}$, ${∥\cdot ∥}_{0}$ and ${∥\cdot ∥}_{0,\Gamma_{v}}$ being norms on $H^{1}\left(\Omega \right)$, $L^{2}\left(\Omega \right)$, and $L^{2}\left(\Gamma \right)$, respectively (c.f (Girault et al., 2001)).

In the current paper, we have selected k>0 as a constant, making λ no longer a Lagrange multiplier and instead a penalty term, relaxing the strict equality between measured and computed flow fields when excessive energy is required to drive the flow. The added variable is then defined by the condition:

(5) $$\left(v-v_{d}\right)-\frac{1}{k}\lambda =0$$

In the simulations presented in this paper the value of k was set to 30,000 Ns/m. This value was chosen based on the ratio between the approximate physiological pressure values and the order of magnitude of the velocity error associated with the image acquisition process.

#### 2.4.2 Penalized Navier–Stokes formulation

The well-posedness of the augmented problem was previously demonstrated in the case of the stationary Navier–Stokes equations (Formaggia et al., 2003). In the unsteady non-conservative ALE Navier–Stokes the momentum and mass conservation laws can be expressed as:

(6) $$\begin{array}{ccc} \rho \partial_{t}\left(v\right)+\nabla_{x}\cdot \left[\rho \left(v-w\right)v-\sigma \right]+\lambda \delta_{\Gamma_{v}}=0 & or & \rho \frac{\partial v_{i}}{\partial t}+\frac{\partial }{\partial x_{j}}\left[\rho \left(v_{j}-w_{j}\right)v_{i}-\sigma_{ij}\right]+\lambda_{i}\delta_{\Gamma_{v}}=0 \end{array}$$

(7) $$\begin{array}{ccc} \nabla_{x}\cdot v=0 & or & \frac{\partial v_{i}}{\partial x_{i}}=0 \end{array}$$

Here, ρ is the fluid density and the vectors v and w are the velocities of the blood and of the ventricular domain, respectively. The ALE time derivative, $\partial_{t}$, tracks the changes in time with respect to the reference frame moving with the ventricle (Nordsletten et al., 2008). The Cauchy stress tensor is based on the Navier–Poisson law, $\sigma =-pI+\mu \left[\nabla_{x}v+v\nabla_{x}\right]$, where *p* is the pressure and μ is the fluid viscosity. The added variable, λ, is defined on the inlet and outlet boundaries by the Kronecker delta $\delta_{\Gamma_{v}}$, which is 1 on the valve planes $\Gamma_{v}$ and 0 elsewhere. The system is then discretized and solved in conjunction with Eq. (5) and the Dirichlet boundary conditions from the imaging data. These are expressed as $\begin{array}{ccc} v=v_{d} & on & \Gamma_{i} \end{array}$, where $\Gamma_{i}$ is the selected boundary, i.e. the endocardial wall and the valve planes. Blood is treated as an incompressible Newtonian fluid with density ρ = 1025 kg/m^3^ and viscosity μ = 0.004 Pa s.

The strong form of Eqs. (5)–(7) is then reduced to the weak form and discretized over the spatial domain for each time step using finite elements interpolation (Appendix A).

## 3 Results

The proposed implementation is first applied to simulate inflation and deflation in an idealized two-dimensional half-ellipse. To test the stability of the approach, different levels of Gaussian noise are added to the boundary conditions and the results are compared to the non-perturbed solution. Different values of the parameter k are also tested. The methodology is then applied to model a patient with mitral stenosis after surgical repair of the aortic valve.

### 3.1 Validation of the numerical method: the idealized half-ellipse

**Fig. 2:**
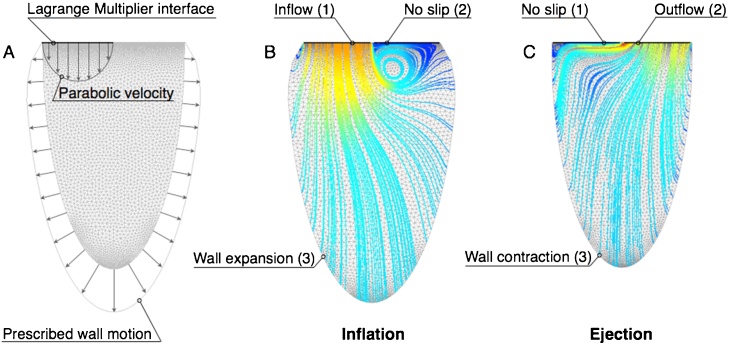
(A) two-dimensional half-elliptical mesh with prescribed wall motion and parabolic flow velocity at the valve. (B–C) Boundary conditions for the simulation of inflation and ejection.

The numerical method is tested in a triangular mesh with 10,313 elements (51,017 degrees of freedom) of a two-dimensional ellipse subjected to prescribed inflation and deflation. The location of the boundary conditions and the corresponding patches are illustrated in Fig. 2 and can be easily extended to real patient anatomies. In this idealized case, however, the inlet and outlet of the half-ellipse are considered fixed, unlike in the patient models, where each valve plane has a prescribed motion in addition to the inflow and outflow velocities.

Parabolic velocity profiles are imposed at the inflow and outflow to match the volume change due to the prescribed wall motion (Fig. 2A). Two snap-shots in time showing the velocity streamlines and the imposed boundary conditions during the inflation and ejection phases are shown in Fig. 2B–C, respectively. The added variable is defined on the boundary patch where the velocity profile is prescribed, i.e. patch 1 in inflation and patch 2 in ejection, while a no-slip condition is assigned to the other valve plane. The stability of the method is subsequently tested. Specifically, additional simulations are performed by perturbing the initial boundary conditions on the wall velocity without altering the threshold value for the constant k, which remains fixed at 100,000 Ns/m. Three levels of normally distributed noise with zero mean and magnitude equal to 5%, 7% and 9% of the original velocity are tested. The resulting solution for the velocity $v_{n}$ is compared to the original field v computed in the absence of noise. The discrepancies are quantified by the *L*^2^ norm of the difference $v-v_{n}$ computed throughout the duration T of the cardiac cycle. This is then normalized by the norm of the unperturbed maximum velocity, i.e. ${\left\|v\right\|}_{\infty }=\max\left\{\left\|v(t)\right\|,\forall t\in \left[0,T\right]\right\}$, to give relative *L*^2^ error, e(t):

(11) $$e(t)=\frac{\left\|v(t)-v_{n}(t)\right\|}{\|{v\|}_{\infty }}$$

**Fig. 3:**
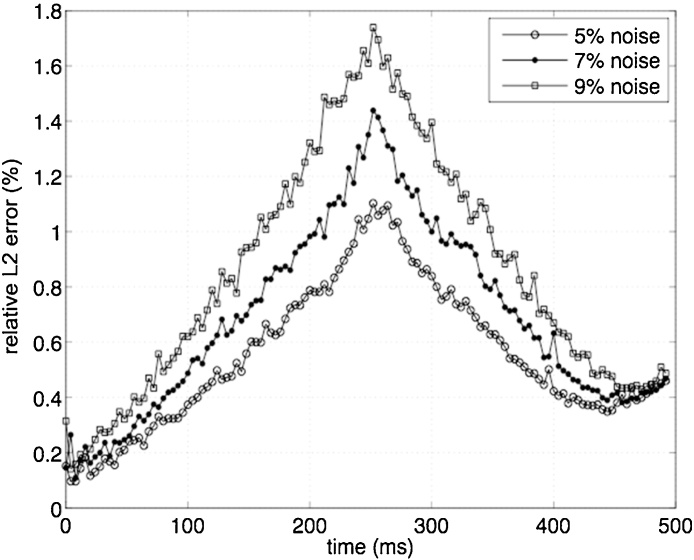
Relative *L*^2^ error in inflation (0–250 ms) and ejection (251–495 ms) between unperturbed and perturbed solution with three different levels of noise in the wall velocity.

As shown in Fig. 3, the percentage of error is below 2% for all perturbation levels.

**Fig. 4:**
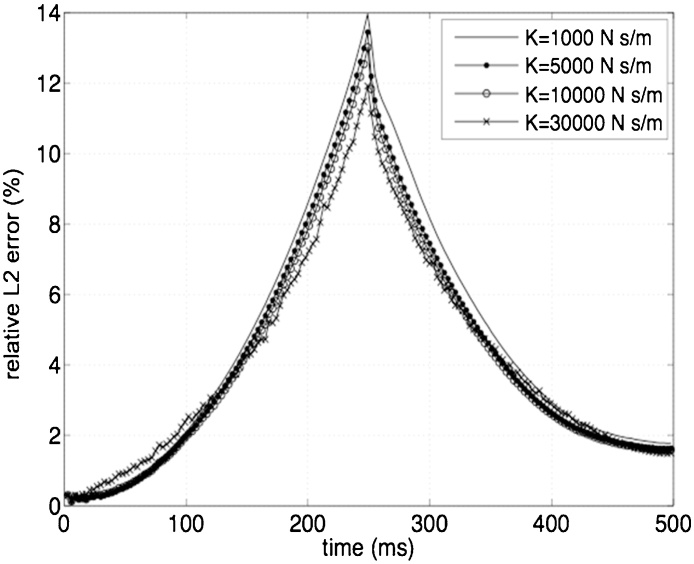
Relative *L*^2^ error between the solution with different values of *k* and a noise level of 9%, and the reference solution without noise.

To assess the effect of the relaxation parameter on the results, simulations are also performed at the highest noise level (9%) using four different values of k, i.e. 1000, 5000, 10,000 and 30,000 Ns/m (Fig. 4). These results are compared to the reference solution v obtained in the absence of noise with k = 100,000 Ns/m. The relative *L*^2^ error is then calculated in each case as:

(12) $$e_{i}(t)=\frac{\left\|v(t)-v_{n_{i}}(t)\right\|}{{\left\|v\right\|}_{\infty }}$$

where $v_{n_{i}}$ represents the solution for each i the value of k and 9% noise in the boundary conditions. The error $e_{i}$ does not show a significant variability with different levels of the parameter k, with a maximum value of 14% for the lowest level of k tested (1000 Ns/m) and a minimum of 12% for k = 30,000 Ns/m (Fig. 4).

### 3 2 Case study: left ventricle with mitral stenosis

#### 3.2.1 Model generation

After testing the stability and robustness to noise of the numerical method, the modeling workflow is applied to the left ventricle of a 2-months old patient with stenosis of the mitral valve. The patient presents normal left ventricular morphology, with a heart rate of 97 beats per minute and ejection fraction above 50%. The personalized mesh of the ventricle consists of 103,393 tetrahedral elements, corresponding to approximately 500,000 degrees of freedom.

#### 3.2.2 Numerical simulation of the cardiac cycle

**Fig. 5:**
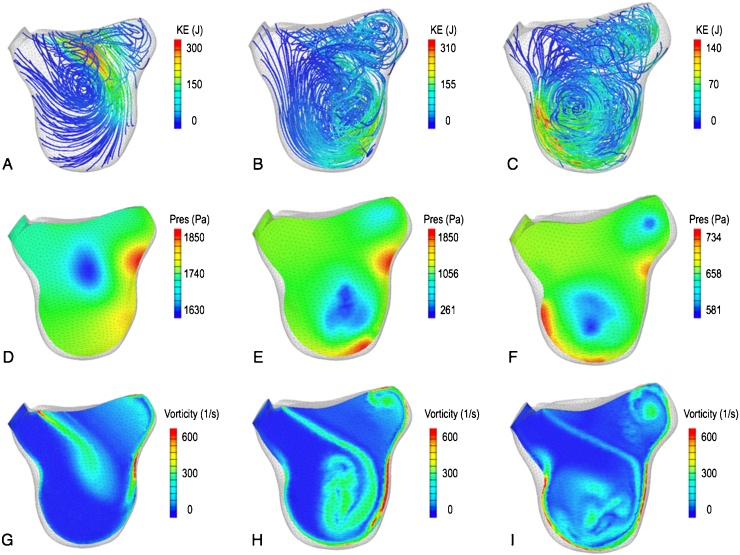
Time snapshots of the vortex formation dynamics in the acceleration phase (left column), at peak E-wave (central column) and in the deceleration phase (right column). (A–C) Blood streamlines colored by the total kinetic energy. (D–F) Iso-contours of pressure. (G–I) Iso-contours of vorticity.

The full cardiac cycle is simulated with the time-varying boundary conditions that are obtained by interpolating the motion and velocity derived from the imaging data to a temporal resolution of 1 ms. The main diastolic feature is the formation of a vortex of blood during the acceleration phase of the inflow, as shown through the blood streamlines colored by the kinetic energy in Fig. 5A–C. This well-documented feature (Kilner et al., 2000, Pedrizzetti et al., 2014) is associated with a region of low pressure located approximately in the center of the vortical motion (Fig. 5D–F). The highest values of kinetic energy are initially located at the inlet plane and start to shift towards the apical region during the deceleration phase after the maximum inflow velocity is reached (peak E-wave). Fig. 5G–I shows the shear layers that form the vortex through iso-contours of vorticity magnitude. The significant difference in size between each side of the shear layers is due to the asymmetry in the formation dynamics, a typical feature of the left ventricular diastolic vortex that in this case is exacerbated by the skewed inflow from the stenotic mitral valve.

**Fig. 6:**
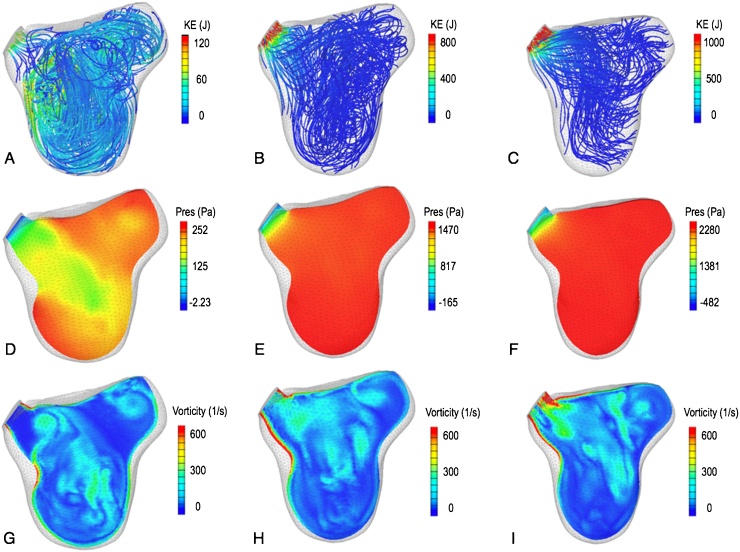
Time snapshots of ejection at the opening of the aortic valve (left column), and early (central column) and mid systole (right column). (A–C) Blood streamlines colored by the total kinetic energy. (D–F) Iso-contours of pressure. (G–I) Iso-contours of vorticity.

The physical mechanisms that occur during systole are shown in Fig. 6. Due to the high gradient in the outflow velocity, the mesh in the region near the aortic valve was systematically refined and the time step was halved in order to achieve a converged solution. At the aortic valve opening the hemodynamics appear chaotic, with the breakdown of the diastolic vortex reflected in the absence of a distinct region of low pressure and of a coherent distribution of vorticity (Fig. 6A,D,G). In the later stages of systole the strong pressure gradient initiated by contraction re-aligns the blood streamlines towards the aortic valve (Fig. 6B–C and E–F). This process is associated with localized regions of high vorticity magnitude near the aortic root (Fig. 6H–I) and with a kinetic energy peak at the outflow tract (Fig. 6B–C).

#### 3.2.3 Effect of temporal and spatial resolution of the imaging data

**Fig. 7:**
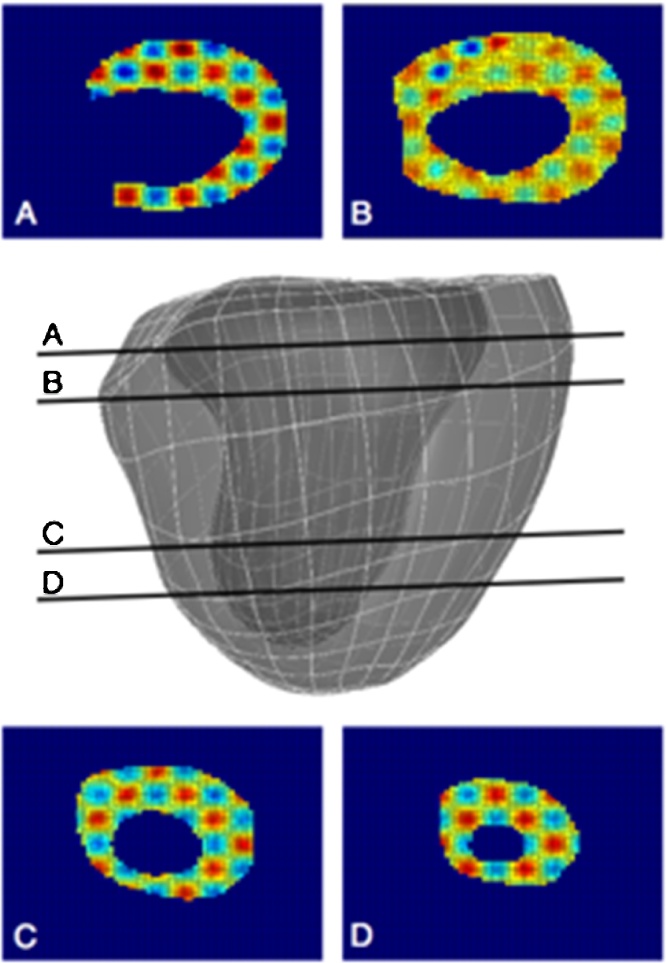
Model of the myocardium used for the generation of the synthetic dataset. (A–D) Slices of the texturized synthetic image derived from the numerical model.

In the previous section, the results from the insilico model are obtained and validated based on one clinical dataset and the corresponding acquisition parameters. To test the robustness of this modeling technique, the stability of the workflow is also investigated with respect to different levels of noise and spatiotemporal resolution of the images that are typical of the acquisition process. To this end, a synthetic imaging dataset is generated from the numerical results in the patient case following a three-step procedure. First, a model of the whole myocardium is obtained by morphing a template mesh to the segmented data (Lamata et al., 2011), as shown in the central part of Fig. 7. This myocardial mesh is then deformed using the same wall motion of the original simulation, with a time resolution of 1 ms. Finally, each time frame of the numerical results is transformed into a 3D synthetic image, whose cross-sections are shown in Fig. 7A–D. A sinusoidal function f(x,y,z)=A(1.25+sin(ωx)sin(ωy)sin(ωz)) is specified across the myocardium to simulate a texture, thus allowing the TSFFD algorithm to track the texture features of the synthetic myocardium. The constant A and the frequency ω are chosen based on the wall thickness and on the voxel intensity observed in the real images. This high-resolution synthetic dataset is subsequently down-sampled in space and time to reproduce common acquisition settings. The parameter space investigated spans five different time resolutions, i.e., 10, 20, 30, 40 and 50 ms. For each temporal resolution, the voxel size is gradually increased from the original resolution of 0.5 mm^3^ to 0.8, 1.0, 1.5 and 2 mm^3^. These temporal and spatial resolutions enclose the range of feasible resolutions in clinical echocardiographic and MR images. This generates a parameter space of 20 space-time combinations. The tracking algorithm is then applied to recover the wall motion for each case. A 15% noise level is also added to the computed wall velocities to investigate the effect of perturbations in the data acquisition. The noise was chosen to be additive white Gaussian noise with standard deviation of 2.75 to reproduce a random perturbation with zero temporal mean, which approximates the noise distribution in bright regions of MRI images such as the blood pool (Bao and Zhang, 2003). Finally, simulations of the cardiac cycle with and without the added noise are performed with the new sets of boundary conditions. For each case, the perturbed results are compared to the original simulation to quantify the *L*^2^ error in the velocity field, as expressed in Eq. (11).

**Fig. 8:**
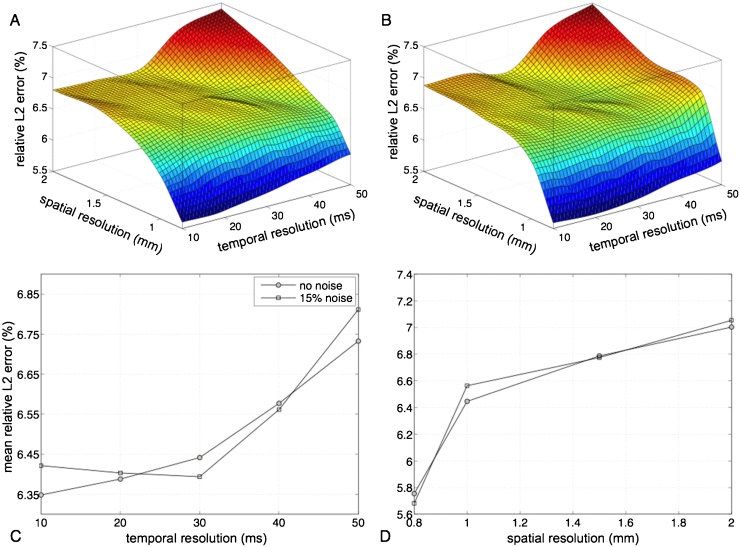
Relative *L*^2^ error between the original velocity field and the one computed at different spatial and temporal resolution without added noise (A) and with added noise (B). (C–D) Mean relative *L*^2^ error for each temporal and spatial resolution, respectively.

The combined effect of common sources of error in the image acquisition on the accuracy of the velocity calculations is subsequently investigated, as well as the robustness and reproducibility of the numerical results. The nonlinear behavior of the *L*^2^ error in the parameter space is presented in Fig. 8A,B for the simulations without and with the added noise, respectively. The mean error in the case without noise is lower than in the case with noise (6.49 ± 0.51 vs 6.52 ± 0.56). In both cases the error increases monotonically with decreasing resolution in space and time. However, reducing the spatial resolution results in a higher error increase than that observed when the temporal resolution is lowered, both with and without the addition of noise. The *L*^2^ error averaged in space and time exhibits a variance of 0.02 (0.03 with noise) when the temporal resolution is varied (Fig. 8C) and 0.29 (0.35 with noise) when the spatial resolution is varied (Fig. 8D). The mean errors relative to variations in temporal resolution are 6.49 ± 0.16 (without noise) and 6.51 ± 0.18 (with noise), while the values relative to variations in spatial resolution are 6.5 ± 0.55 (without noise) and 6.52 ± 0.6 (with noise). Finally, the addition of noise does not affect the velocity computations significantly, with a maximum error magnitude below 10% for both perturbed and unperturbed results, indicating that the workflow is robust to noise.

## 4 Discussion and conclusion

This paper presents an image-based framework for ventricular hemodynamics simulations. The methods was first validated in a 2D idealized ellipse, then applied to a patient case as a proof of concept. Synthetic datasets were also generated from this case to investigate the robustness of the numerical results. The main features of this technique are focused on widening its clinical applicability and are: (1) the use of standard imaging datasets, without the need for invasive measurements for the model personalization or more sophisticated acquisition sequences; (2) the application of a penalty method to the weak form of the Dirichlet boundary condition on image-derived velocity; (3) the robustness to noise and sub-optimal spatiotemporal resolution of the imaging data. The proposed approach aims at providing an efficient methodology for integrating the progress that is being independently achieved by image processing and numerical modeling techniques. PC-MRI and 3D+t echocardiography data can now provide intraventricular blood flow mapping in space and time, with a view to enhance the diagnosis and clinical decision-making process. Recent studies have shown that global blood flow dynamics can provide early indication of pathophysiological processes since flow is immediately affected by alterations in cardiac function (Pedrizzetti and Domenichini, 2014, Pedrizzetti et al., 2014). Specifically, intraventricular vortical motion is significantly modified by the inception and progression of diseases such as dilated cardiomyopathy, thrombus formation or valvular pathologies (Bermejo et al., 2015, Carlhäll and Bolger, 2010, Faludi et al., 2010, Son et al., 2012). The quantitative analysis of intraventricular flow parameters such as kinetic energy, pressure gradients and hemodynamic efficiency has also potential to identify risks of adverse remodeling before irreversible maladaptations take place in the ventricular architecture (Sengupta et al., 2012). How this potential can be put into a clinically meaningful context through the derivation of new biomarkers for specific pathologies is however still open to question. Our approach delivers a tool based on wall motion tracking techniques that have been extensively validated and are now able to provide an accurate reconstruction of the myocardial velocities. Numerical models based on these data have the capability to complement and enhance the quantitative flow parameters derived from imaging data. Until now, this potential has not been fully exploited. The sole imposition of Dirichlet boundary conditions from image-derived velocities poses numerical difficulties that have hindered the clinical translation of this type of approach so far. The necessity of using invasive pressure data for the models’ personalization has also limited the number of patients that can potentially benefit from the approach. The proposed methodology combines a penalty based numerical implementation of the boundary conditions with the use of well-established imaging processing techniques. The parameter k in the augmented formulation of the Navier–Stokes problem reflects the confidence in the image-derived data used to drive the numerical simulations. In the case study we have chosen to fix the value of k, making this approach akin to a penalty method. However, a future direction of research could include the implementation of a spatially varying k, which can potentially send 1/k to zero, implying that the added variable λ becomes a Lagrange multiplier instead of a penalty term.

To our knowledge, this is the first time a computational toolbox integrates processing of multiple imaging modalities and penalty methods to produce image-driven simulations of ventricular hemodynamics. This integration also allows a faster, more efficient model generation process that aims at accelerating the clinical translation of the approach. This is underpinned by the flexibility of the workflow, which is not restricted to a specific imaging technique, therefore extending its applicability to retrospective, as well as prospective, clinical studies. Sensitivity analysis in a controlled environment provided by synthetic datasets has showed that the method is robust with respect to variations in levels of noise and spatiotemporal resolution of the input imaging data. This point is of particular relevance as it provides a quantitative measure of the margins of errors inherent in the image-based modeling process, which is an essential step towards a full clinical translation of this approach. However, although numerical modeling now allows for a comprehensive representation of ventricular hemodynamics, several limitations should be discussed with respect to the clinical applicability of this technique. The spatial resolution is dictated by the characteristics of the computational mesh, which can be refined ad hoc in regions where accuracy is important. Temporal resolutions can also be increased to 1 or 2 ms. This allows us to compute spatial and temporal derivatives of the velocity field with high accuracy to derive clinical metrics such as wall shear stress and viscous dissipation. However, since the current model does not account for the presence of trabeculations and roughness of the endocardial surface, a reliable quantification of the wall shear stress in the ventricular cavity cannot be achieved and the viscous energy loss due to friction at the wall is likely to be underestimated in a smooth model. Similarly, local flow dynamics such as myocardial blood perfusion and coronary blood flow cannot be simulated in the current approach. While such quantitative measurements can be inferred from advanced imaging modalities such as dynamic CT, it is not clear whether the added diagnostic benefits can outweigh higher scanning times and radiation doses (Saraste and Knuuti, 2015). These are obvious limitations of any computational model that does not include the myocardium, and therefore inherent to any hemodynamic model like the one presented here. The proposed workflow can however be applied to model vessels, where the smooth walls and the absence of obstacles (with the exception of specific pathologies), make the wall shear stress and power loss calculations more meaningful and accurate. Although this application lies beyond the scope of the present study, it opens an avenue of investigation well worth exploring, both with respect to the modeling of single vessels and of coupled arterio-ventricular systems. However, these limitations cannot be currently overcome with imaging data analysis either due to the technical difficulties to quantify the blood velocity near the wall, where the spatial resolution of the images poses significant limitations to the accuracy of both the velocity and its derivatives. Finally, due to the nature of the boundary conditions, the models can quantify metrics of clinical importance such as the intraventricular pressure gradients but not the absolute pressure, whose value can be derived from the computed results only if a direct pressure measurement is available.

## Conflict of interest

None.

## Appendix A . Discretization of the Navier–Stokes system

The system of Eqs. (5)–(7) is cast into weak form and discretized over the fluid domain $\Omega_{f}$ for each time interval $T_{n}=\left[t^{n-1},t^{n}\right]$. The ${\mathbb{P}}^{2}-{\mathbb{P}}^{1}$ Taylor–Hood basis functions, denoted over the mesh by $\left\{\psi_{v}^{1}\ldots \psi_{v}^{N_{v}}\right\}$ and $\left\{\psi_{p}^{1}\ldots \psi_{p}^{N_{p}}\right\}$, were used to construct the velocity field and the pressure field, respectively (Brezzi and Falk, 1991, Nordsletten et al., 2010a, Nordsletten et al., 2010b, Nordsletten et al., 2008). The weak formulation of the ALE Navier–Stokes problem can thus be expressed as:

(A.1) $$\rho \int_{\Omega_{f(t^{n})}}v^{h,n}\cdot \psi_{v}^{k}dx-\rho \int_{\Omega_{f(t^{n-1})}}v^{h,n-1}\cdot \psi_{v}^{k}dx+\rho \int_{T_{n}}\int_{\Omega_{f(\tau )}}\left[\nabla_{x}\cdot \left[(v^{h,n}-w^{h,n})v^{h,n}\right]\right]\cdot \psi_{v}^{k}dxd\tau +\mu \int_{T_{n}}\int_{\Omega_{f(\tau )}}\left[\nabla_{x}v^{h,n}+v^{h,n}\nabla_{x}\right]:\nabla_{x}\psi_{v}^{k}dx\text{d}\tau -\int_{T_{n}}\int_{\Omega_{f(\tau )}}p^{h,n}\left[\nabla_{x}\cdot \psi_{v}^{m}\right]dxd\tau +\int_{T_{n}}\int_{\Gamma_{v(\tau )}}\lambda^{h,n}\cdot \psi_{v}^{k}dxd\tau =0$$

(A.2) $$\int_{T_{n}}\int_{\Omega_{f(\tau )}}\psi_{p}^{k}(\nabla_{x}\cdot v^{h,n})dx\text{d}\tau =0$$

for *k* *=* 1, *…*, *N_v_* and *m* *=* 1, *…*, *N_p_*.

The additional constraint imposed on the boundary $\Gamma_{v\left(\tau \right)}$in Eq. (5) can be written as:

(A.3) $$\int_{T_{n}}\int_{\Gamma_{v(\tau )}}\psi_{\lambda }^{k}\left(v^{h,n}-v_{d}-\frac{1}{k}\lambda^{h,n}\right)dxd\tau =0$$

for *k* *=* *1*, …, *N_L_*.where $\left\{\psi_{\lambda }^{1}\ldots \psi_{\lambda }^{N_{L}}\right\}$ are the test functions for the penalty variable and represent the trace of the velocity space on the applied boundary (i.e. ${\mathbb{P}}^{2}$ nodal Lagrange polynomials were used to construct the approximation space on $\Gamma_{v\left(\tau \right)}$). The numerical solution of the discretized system of Eqs. (A.1)–(A.3) is then achieved iteratively by means of a Newton–Raphson method within the finite element software *CHeart* (Nordsletten et al., 2011, Nordsletten et al., 2010b).
